# Gallstones and Concomitant Gastric *Helicobacter pylori* Infection

**DOI:** 10.1155/2013/643109

**Published:** 2013-05-19

**Authors:** Wafi Attaallah, Nese Yener, M. Umit Ugurlu, Manuk Manukyan, Ebru Asmaz, A. Ozdemir Aktan

**Affiliations:** ^1^Marmara University, School of Medicine, Department of General Surgery, 34899 Istanbul, Turkey; ^2^Maltepe University, School of Medicine, Department of Pathology, 34844 Istanbul, Turkey; ^3^Maltepe University, School of Medicine, Department of General Surgery, 34844 Istanbul, Turkey

## Abstract

*Background*. The association of gallstones with *Helicobacter pylori* has been investigated but not clearly demonstrated. In this study, the presence of *H. pylori* in the gallbladder mucosa of patients with symptomatic gallstones was investigated. *Method*. Ninety-four consecutive patients with symptomatic gallstone disease were enrolled for the study. Gastroscopy and gastric *H. pylori* urease test were done before cholecystectomy to all patients who accepted. After cholecystectomy, the gallbladder tissue was investigated in terms of *H. pylori* by urease test, Giemsa, and immunohistochemical stain. *Results*. Overall 35 patients (37%) gallbladder mucosa tested positive for *H. pylori* with any of the three tests. Correlation of the three tests Giemsa, IHC, and rapid urease test was significant (*r*_*s*_: 0590, *P* > 0.001). Rapid urease test was positive in the gastric mucosa in 47 (58.7%) patients, and it was positive in the gallbladder mucosa in 21 patients (22%). In 15 patients both gastric and gallbladder tested positive with the urease test. There was significant correlation of rapid urease test in both of gallbladder and gastric mucosa (*P* = 0.0001). *Conclusion*. Study demonstrates the presence of *H. pylori* in the gallbladders of 37% of patients with symptomatic gallstones.

## 1. Introduction

Gallstone disease is one of the most common problems affecting the digestive tract where autopsy reports show a prevalence of 11–36% [1]. The prevalence of gallstones is related to many factors including age, gender, and ethnic background. Women are three times more likely to develop gallstones than men, and first-degree relatives have a twofold increased prevalence [2]. However, the etiology of gallstone formation, beginning with a change in the composition of bile, leading to stones, is not clear.

The association between *Helicobacter pylori* (*H. pylori*) and gallstones has been investigated but not clearly demonstrated. *H. pylori* is a Gram negative and microaerophilic microorganism that can cause chronic gastritis, gastric and duodenal ulcers, gastric and pancreatic adenocarcinoma, and lymphoma of gastric mucosa-related lymphoid tissue (MALToma) [3–9]. The relationship of *H. pylori* with diseases of organs other than the stomach and duodenum has also been investigated and reported [10, 11]. Antibodies to *H. hepaticus*, often cross reacting with *H. pylori*, were detected in patients with chronic liver diseases [12]. Also, *H. pylori* have been detected in the gallbladder mucosa of patients with gallstones [13].

In this study, the presence of *H. pylori* in the gallbladder mucosa of patients with symptomatic gallstones undergoing cholecystectomy was investigated. Concomitant *H. pylori* infections of the gastric mucosa were also investigated to study the relationship of gastric *H. pylori* infections to gallstones. It was hypothesized that *H. pylori* infection of the gastric mucosa may have a role in the formation of gallstones.

## 2. Material and Methods

The study was conducted on patients undergoing laparoscopic cholecystectomy for symptomatic cholelithiasis in Marmara University Hospital and Maltepe University Hospital, Istanbul, Turkey. The Research Ethics Committee approved the study (approval number: B.30.2.MAR.0.01.02/AEK/73), and all patients signed the informed consent.

### 2.1. Study Group

Ninety-four consecutive patients (31 male, 63 female; mean age 48) with symptomatic gallstone disease were enrolled for the study. Patients with acute cholecystitis, cholangitis, biliary and hepatic tumors, Crohn's disease, and previous gastric surgery were not considered suitable for evaluation. Patients undergoing ERCP (endoscopic retrograde cholangiopancreatography) and patients who had received *H. pylori* eradication treatment in the last 6 months were also excluded from the study. Gastroscopy and the gastric *H. pylori* urease test (Pronto Dry) were done before the surgical procedure for all patients who accepted.

### 2.2. Determination of *H. pylori* Status

After laparoscopic cholecystectomy, the gallbladder was opened in the operating room, and three strips of tissue were obtained from the infundibulum of the gallbladder. One sample was used for the rapid urease test (Pronto Dry), the second for aerobic and anaerobic culture, and the third for histopathologic evaluation. Gallstones were classified as cholesterol, pigment, and mixed stones based on their color and consistency [14, 15].

In those patients who had accepted to undergo gastroscopy prior to their laparoscopic cholecystectomy, biopsy specimens were collected from the antrum of the stomach, and the *H. pylori* status was determined using the rapid urease test (Pronto Dry).

### 2.3. Gallbladder Processing

**Figure 1:**
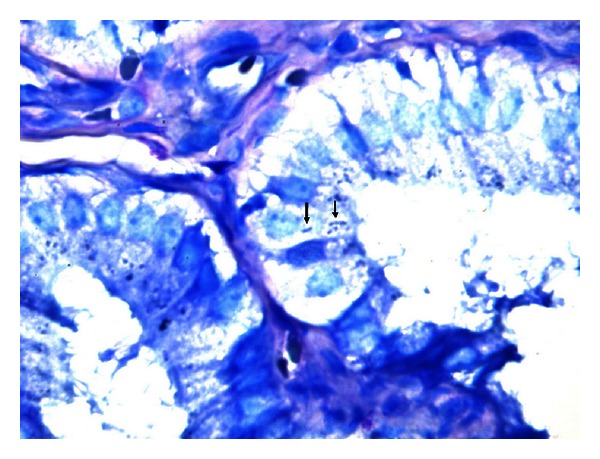
*H. pylori* within the gallbladder mucosa epithelium (MGG, ×1000).

**Figure 2:**
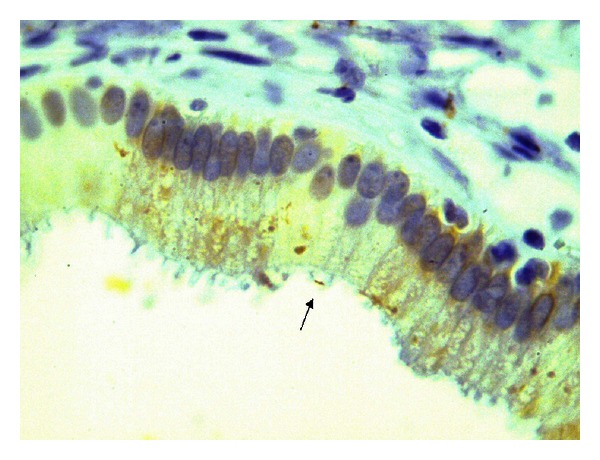
Immunopositivity for *H. pylori* along with the gallbladder mucosal columnar epithelium. Note that the bacilli are noninvasive and placed on the apical surface of the epithelium (×400).

For histopathologic examination specimens were fixed in 10% buffered formalin and stained with a modified Giemsa stain. Under light microscopy curved, bent, pole-like, spiral, and fusiform bacteria were accepted as *H. pylori*-like bacteria (Figure 1). Immunohistochemical studies were also carried out on the fixed specimens (Figure 2). Tissue sections were placed on poly-L-lysine coated slides, and *H. pylori* antigen was determined according to the manufacturer's instructions (GeneTex, GTX 74404, San Antonio, TX, USA). Immunoreactivity was recorded as positive or negative.

### 2.4. Statistical Analysis

Relationships between variables were tested by the Pearson and Spearman correlation coefficients (Giemsa, IHC, and rapid urease test), and McNemar's test was used to compare the rapid urease test in the gallbladder mucosa and the rapid urease test in the gastric mucosa; *P* < 0.05 was considered as statistically significant.

## 3. Results

Ninety-four patients with symptomatic gallstones undergoing laparoscopic cholecystectomy were enrolled for the study. Eighty of these patients had agreed to undergo gastroscopy before surgery. The rapid urease test in the gastric mucosa was negative in 33 (41.3%) and positive in 47 (58.7%) of these patients. The rapid urease test was positive in the gallbladder mucosa of 21 patients (22%). In 15 patients both gastric and gallbladder mucosa tested positive with the urease test. In 26 patients the gastric urease test was positive while the gallbladder mucosa tested negative. In only two patients the gallbladder mucosa tested positive while the gastric mucosa was negative. In four patients with a positive urease test in the gallbladder mucosa a gastric endoscopy was not done due to the patient's refusal. There was a significant correlation between the rapid urease test in the gallbladder mucosa and in the gastric mucosa (*P* = 0.0001, Table 1).

Laparoscopic cholecystectomy was completed uneventfully in all 94 patients with no serious postoperative complications. On histopathologic examination after Giemsa staining, *H. pylori*-like bacteria were detected in 25 patients (27%), and with immunohistochemical study, *H. pylori* antibodies were detected in 17 (18%). In 12 patients all three tests were positive. Urease positivity alone was seen in 8 patients, while Giemsa staining was the only positive test in 10 patients. Immunohistochemical positivity as the only positive test was seen in only one patient. Overall in 35 patients (37%) gallbladder mucosa tested positive for *H. pylori* with any one of the three tests. Correlation of three tests Giemsa, IHC, and rapid urease test was significant (*r*_*s*_: 0.590, *P* < 0.001, Table 2). Twenty-four patients had cholesterol stones (25.5%), 32 had pigment stones (34.5%), and 38 (40%) had mixed stones. There was no significant relationship between stone type and *H. pylori*. Cultures taken from the gallbladder mucosa grew microorganisms in 38 patients (40%). 13 different microorganisms were isolated, *E. coli* being the most common. 

## 4. Discussion

Overall, in 35 of 94 patients undergoing cholecystectomy for symptomatic gallbladder stones, the gallbladder mucosa tested positive for *H. pylori* with any one of the three tests used in this study. This positivity was commonly associated with the presence of *H. pylori* in the gastric mucosa.

Detection of the presence of *H. pylori* in bile can be done with different methods, which are far from being perfect. The best way to show *H. pylori* is to grow *H. pylori* in cultures, but *H. pylori* are extremely hard to culture due to the microaerophilic properties of this microorganism, which die when they contact air. In another study it was shown that various PCR techniques could be used as a method for detection of *H. pylori* DNA in bile [16]. Fallone et al. [17] failed to find DNA of the genus *Helicobacter* in the bile of Canadian patients with biliary disorders, whereas a completely contrary result was described by Silva et al. (who found bacterial nucleotide sequences in most Brazilian subjects with similar diseases) [18]. Regional differences due to variable rates of infection and the changing sensitivity of the various PCR techniques used may be responsible for the difference in the reported studies [19].

The rapid urease test is easy to use and reliable. This test was positive in 58.7% of the gastroscopies performed in our study. In 15, the urease test was positive in both the gastric and gallbladder mucosa. On the other hand, of 47 patients with *H. pylori* in the stomach, the gallbladder showed the presence of *H. pylori* in 20 patients who tested positive with any one of the three tests used in this study (43%) revealing the close relationship of the common presence of *H. pylori* in both organs. Gallbladder mucosa in only two patients tested positive for the urease test while the stomach was negative for *H. pylori*. No signs of *H. pylori* were detected in the gallbladder in 59 of the 94 patients operated.

With the Giemsa stain, *H. pylori*-like bacteria were found in 27% of 94 gallbladder specimens while *H. pylori* antibodies were detected in 18% by immunohistochemistry. Chen et al., using W-S silver stain and light microscopy, showed *H. pylori*-like bacteria in 13.6% of cholecystectomy specimens, while only 7.1% tested positive by immunohistochemistry [13]. Detection of antibodies is important because this test is specific for *H. pylori*, and the other two tests used in this study also detect *H. pylori*-like organisms. With the Giemsa stain, *H. pylori*-like organisms were mainly seen on the surface of epithelial cells of the gallbladder mucosa and uncommonly in the intercellular zone or within the mucous gland. The *H. pylori* were seen as spiral, U- and S-shaped in morphology and were distributed in a scattered or aggregated fashion. All these characteristics are similar to those of *H. pylori* in the stomach.

Previous studies with different methods have revealed the presence of *H. pylori* in the biliary tract in 50–60% of patients studied [20–22]. *H. pylori* in the gastric mucosa is much more common. In Turkey and similar countries *H. pylori* is found in 80% of the population [23]. Our study and others clearly demonstrate that *H. pylori* can resist bile salts and can survive and colonize in the biliary tract. The route of infection, however, is not clear.

Monstein et al. have demonstrated *H. pylori* DNA in cholesterol gallstones and claimed the role of *H. pylori* in the etiology of cholesterol gallstones [8]. *H. pylori* infection of the gallbladder has been shown to increase the precipitation of cholesterol to form stones [24, 25]. Also it has been shown that urease induced calcium precipitation by *Helicobacter* species that may initiate gallstone formation [26]. We think that chronic *H. pylori* infection of the gallbladder may impair gallbladder contractility and so lead to increase in the precipitation of bile components to form stones. In our study no relationship was found for the presence of *H. pylori* in the gallbladder and the type of the gallstone.

This study demonstrates the presence of *H. pylori* in the gallbladder in 37% of patients with symptomatic gallstones. This study also demonstrates the concomitant presence of *H. pylori* in the gastric and gallbladder mucosa. However, it does not suggest *H. pylori* for the etiology of gallstones. Nevertheless, the effect of *H. pylori* eradication on the incidence of gallstones remains to be investigated.

## Figures and Tables

**Table 1:** Rapid urease test results in gastric mucosa and gallbladder mucosa.

Rapid urease test	*H. pylori* positivity *N* (%)	*H. pylori* negativity *N* (%)	*P* value
Gastric mucosa	47 (58.7%)	33 (41.3%)	*P* = 0.0001
Gallbladder mucosa	21 (22%)	71 (78%)	
Both	15 (19%)	NA	NA

**Table 2:** Detection of *H. pylori* presence with different tests.

Tests	*H. pylori* positivity *N* (%)	*H. pylori* negativity *N* (%)	*P* value
Rapid urease test	21 (22%)	73 (78%)	
IHC	17 (18%)	77 (82%)	*P* = 0.0001 *r*_*s*_ = 0.590
Giemsa staining	25 (27%)	69 (73%)	
Triple positivity	12 (13%)	NA	NA
